# Impact of AI-Triaged Worklists and AI-Assisted Report Generation on Radiology Turnaround Times: Prospective Real-World Study

**DOI:** 10.2196/92181

**Published:** 2026-08-31

**Authors:** Srinath Sridharan, Nicholas Png, Alicia X H Seah, Narayan Venkataraman, Kelvin S H Sng, Wee Kiong Lim, Han Leong Goh, Yan Xian Goh, Andy Ta, Kang Min Wong, Weien Chow, Kee Chong Ng, Charlene Liew

**Affiliations:** 1 Data Science & Intelligence Changi General Hospital Singapore, Singapore Singapore; 2 Department of Vascular and Interventional Radiology Singapore General Hospital Singapore Singapore; 3 Data, AI & Innovation Synapxe Pte Ltd Singapore Singapore; 4 Department of Radiology Changi General Hospital Singapore Singapore; 5 CDDO Division & Department of Cardiology Changi General Hospital Singapore Singapore; 6 Changi General Hospital Singapore Singapore; 7 Department of Biomedical Engineering National University of Singapore Singapore Singapore

**Keywords:** artificial intelligence, AI, workflow, radiography, thoracic, radiology information systems, time factors

## Abstract

**Background:**

Radiology departments frequently manage large, heterogeneous worklists using first-in, first-out (FIFO) reporting workflows. This approach does not account for clinical urgency and may contribute to prolonged reporting delays, particularly in high-volume settings. AI systems are increasingly being integrated into radiology workflows, not only for image analysis but also as tools for worklist prioritization and report generation. However, real-world evidence of their operational impact remains limited.

**Objective:**

This study aimed to evaluate the impact of an AI-triaged reading worklist combined with AI-assisted report generation on radiologist workflow efficiency, measured by report generation time (RGT) and overall turnaround time (TAT) for chest radiographs in a real-world hospital setting.

**Methods:**

We conducted a single-center prospective paired study using a single-sequence crossover design. Eight board-certified radiologists interpreted chest radiographs during 2 reporting sessions: an unaided session using standard FIFO worklists and an AI-assisted session using an AI-triaged worklist with integrated report generation tools, separated by a 4-week washout period. Chest radiographs acquired between November 2023 and January 2024 were included. RGT was defined as the time from opening a study to report finalization, and TAT was defined as the time from the start of a reporting session to report finalization. Statistical comparisons were performed using nonparametric tests.

**Results:**

A total of 1054 chest radiographs were included. Median RGT decreased from 2 (IQR 1-4) minutes in the unaided session to 0.53 (IQR 0.22-1.12) minutes in the AI-assisted session (*P*<.001), representing a 73.3% reduction. The largest reduction was observed in radiographs categorized as normal, with median RGT decreasing from 2 (IQR 1-3) minutes to 0.2 (IQR 0.13-0.35) minutes (a 90% reduction). Mean TAT decreased from 876.21 (SD 1014.20; 95% CI 816.06-940.55) minutes to 82.25 (SD 83.38; 95% CI 76.98-87.27) minutes with AI assistance, corresponding to a 90.6% reduction. Significant reductions in TAT were observed across all urgency categories, including critical studies (all *P*<.001).

**Conclusions:**

In a real-world clinical setting, the use of AI-triaged worklists and AI-assisted report generation was associated with substantial reductions in RGT and overall TAT for chest radiographs. These findings suggest that AI, when deployed as workflow infrastructure rather than a diagnostic replacement, may meaningfully improve radiology operational efficiency and reporting timeliness.

## Introduction

Radiography is the cornerstone of diagnostic imaging in medical practice worldwide [1,2]. However, its widespread use presents challenges in efficiently managing image interpretation and reporting workflows. Radiologists frequently encounter large heterogeneous worklists, often compounded by unexpected surges in imaging demand, which can strain conventional first-in, first-out (FIFO) reporting workflows and contribute to reporting delays [3].

The escalating demand for radiographic services can lead to delays in reporting, resulting in prolonged turnaround times (TATs) for critical diagnoses [4,5]. Such delays impede patient care and heighten the risk of overlooking urgent findings, potentially compromising patient outcomes. In our hospital alone, radiologists reported 145,063 chest radiographs (CXRs) over a 12-month period ending in April 2024, highlighting the scale at which even modest inefficiencies in workflow design may translate into substantial cumulative delays.

AI integration in radiology represents an important development in the evolution of medical imaging workflows. The application of AI techniques to aid radiologists in image interpretation has a rich history dating back several decades. In 1963, Lodwick et al [6] described one of the earliest computer-aided approaches in radiography. Over the years, significant progress has been made in AI algorithms for CXR analysis, with prior studies reporting improvements in report efficiency and lesion detection [7-13]. Beyond image interpretation, AI has increasingly been explored as a tool for workflow support, including worklist triage and prioritization. Etienne et al [14] suggested that using AI may free human capital for more complex tasks. Fazal et al [15] and Chen et al [16] recommended its use as both a diagnostic and prognostic aid.

Commentaries by Adams et al [17] and Laney and Pontali [18] have alluded to leveraging AI’s ability to rapidly screen normal CXRs. This concept is also supported by Blake et al [19] and Yoon et al [20], who demonstrated reductions in workload without compromising clinical safety. Similarly, Topff et al [21] demonstrated a reduction in the time to diagnosis of incidental pulmonary embolism using AI-automated worklists. Collectively, these studies suggest that the value of AI in radiology may extend beyond diagnostic assistance to include operational improvements in reporting workflows.

Against this backdrop, this study investigates the impact of deploying an AI-triaged reading worklist on workflow efficiency. Specifically, we evaluate changes in report generation time (RGT) and overall TAT for CXRs in a real-world hospital setting.

## Methods

### Study Design and Participants

The analysis used prospective, postmarket, real-world data to assess the effect of an AI-triaged reading worklist with a commercial AI decision support tool, Lunit INSIGHT CXR (version 3.1.5.0; Lunit Inc), integrated into the hospital system via an AI orchestration platform, Augmento (version 1.4.6; DeepTek). The focus of this study was to evaluate the operational impact of this workflow integration on reporting efficiency, rather than to assess the diagnostic accuracy of the AI system. The list of target findings inferred by the Lunit software included atelectasis, consolidation, fibrosis, calcification, nodule, pleural effusion, pneumothorax, pneumoperitoneum, mediastinal widening, and cardiomegaly. We adhered to SPIRIT (Standard Protocol Items: Recommendations for Interventional Trials)-AI guidelines to ensure compliance with reporting standards.

Participants from a multiethnic patient cohort visiting the institution between November 2023 and January 2024 were included as a consecutive series.

A total of 8 board-certified radiologists participated in this study, with varying experience ranging from 9 to 24 (mean 15.2, SD 4.1) years in reporting plain-film CXR. Two radiologists were subspecialty-trained in thoracic radiology, 2 in body imaging, 2 in neuroradiology, 1 in interventional radiology, and 1 in musculoskeletal radiology.

**Figure 1:**
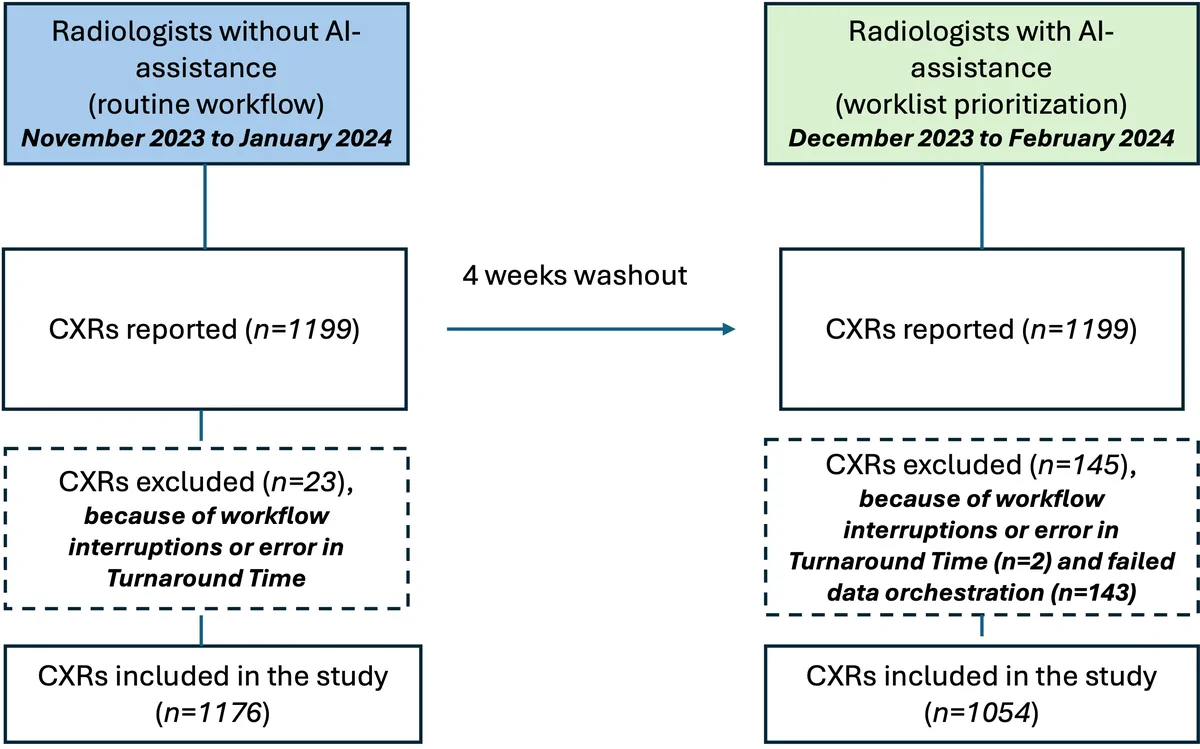
Study design and data selection over the study period. Workflow interruptions or turnaround time (TAT) errors included self-reported interruptions in workflow or technical errors (eg, system downtime). Failed data orchestration was due to nonstandard projections, such as lateral, oblique, and apical chest radiographs (CXRs).

**Figure 2:**
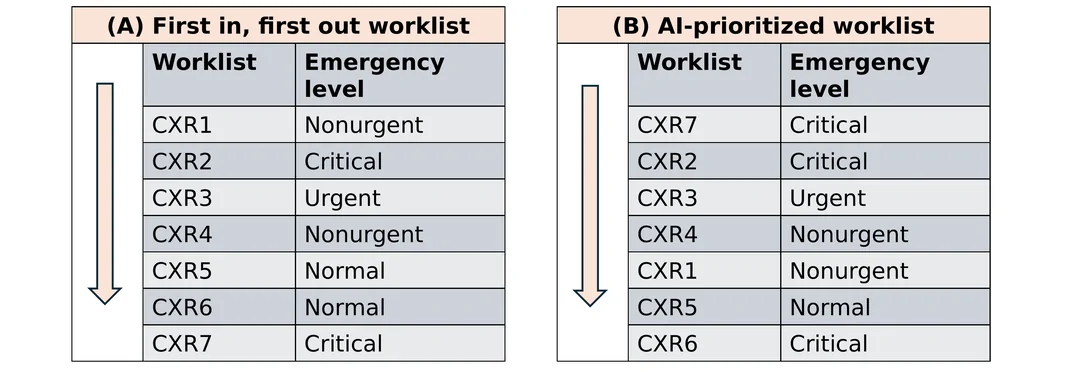
Comparison of worklist prioritization methods. (A) First-in, first-out worklist in session 1 (unassisted) vs (B) AI-prioritized worklist in session 2 (AI-assisted). CXR: chest radiograph.

In the initial phase of the study, radiologists reported real-time plain-film worklists without the use of any AI triage or detection tools from November 2023 to January 2024 (Figure 1), adhering to the routine department workflow. Non-CXRs were filtered out of the worklist to avoid being recorded in this study. Radiologists used the department picture archiving and communication system (PACS), VuePACS (version 12.2.8; Philips). This reference standard workflow followed the “first-in, first-out” sequence for reporting, which reflects routine operational practice but does not differentiate studies by clinical urgency. CXRs were reported in the sequence in which they were sent to the PACS after acquisition (Figure 2).

**Figure 3:**
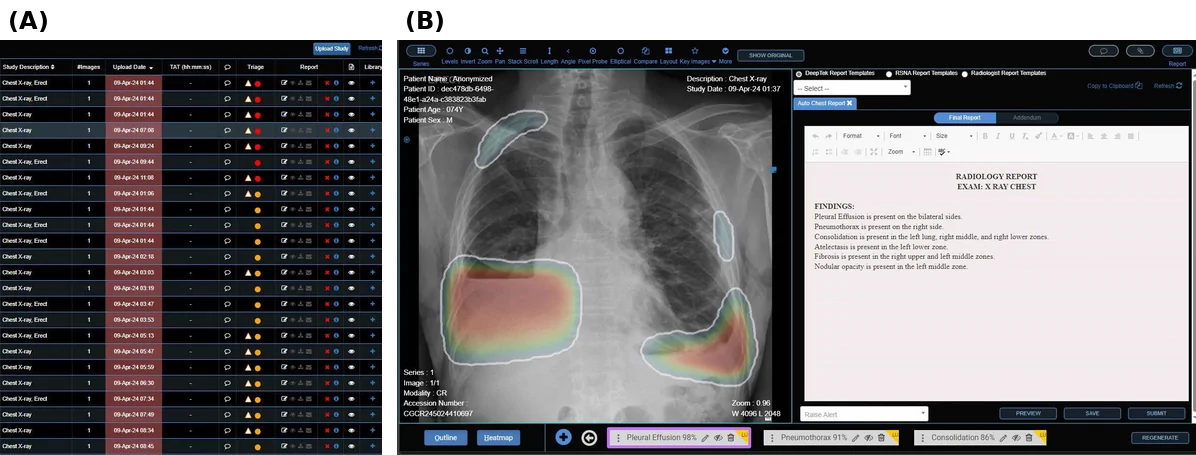
User interface of Augmento (version 1.4.6) with (A) an example of an AI-triaged worklist and (B) the report generation interface with the corresponding heat map.

**Figure 4:**
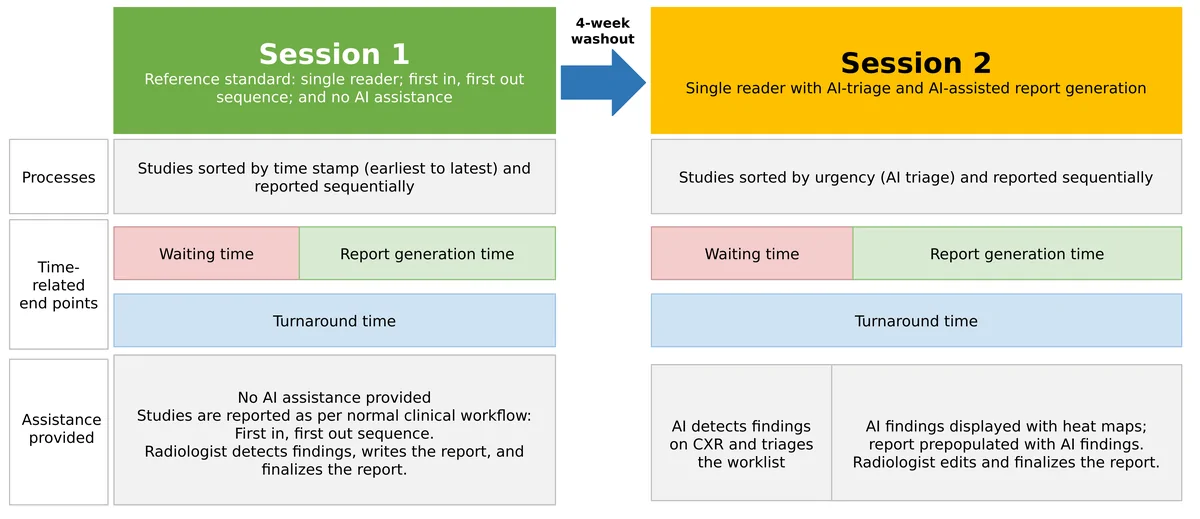
Workflow schematic diagram of session 1 (unassisted) and session 2 (AI-assisted). Waiting time was defined as the time (minutes) between the start of the reporting session and a radiologist opening a study to report. Report generation time was defined as the time (minutes) between a radiologist opening a study to report and report finalization. Turnaround time was defined as the time (minutes) between the start of the reporting session and report finalization. CXR: chest radiograph.

After a 4-week washout period to prevent recall bias, the same radiologists were assigned the same set of CXR studies that had been saved in the AI platform database Augmento and reported them according to the order of triage in the AI-triaged worklist (Figure 3A). This approach confirmed a true within-reader, within-case design. Report generation was assisted by the Augmento report generation tool (Figure 3B) during the second session, and all radiologists were required to use this tool to generate their reports. This tool extracts findings from AI inferences generated by the Lunit INSIGHT CXR AI model and presents each finding as a “smart tag,” together with a heat map and an AI-inferred probability (Figure 3B). Users were allowed to agree or disagree with a finding, and in the end, concordant findings were included in a structured template. Radiologists were also able to edit the free text of the report to add nontarget findings such as catheters and bone lesions. This design reflects routine clinical use of AI-assisted reporting tools under real-world conditions. All radiologists attended a user training session and were given 1 month to familiarize themselves with the system. Figure 4 provides a schematic overview of the workflows in sessions 1 and 2.

Concordance between AI-generated findings and final radiologist reports was analyzed across all 1054 cases. Full agreement was observed in 51.1% (n=539) of cases. Radiologists removed at least 1 AI-suggested finding in 46.7% (n=492) of cases, reflecting active clinical oversight, while they added findings not flagged by the AI in 3.1% (n=33) of cases. Per-finding agreement ranged from 78.2% (n=824) for nodules to 99.1% (n=1044) for pneumothorax and pneumoperitoneum.

All CXR studies reported from November 2023 to January 2024 were included. Exclusion criteria included workflow interruptions due to system downtime from unrelated cloud server issues and nonstandard CXR projections, including lateral, oblique, and apical views (Figure 1).

The reference standard of care was determined by a single read by a board-certified radiologist, unaided by AI models. Correlation with cross-sectional imaging or histopathological correlation was not sought, as this was not representative of a real-world clinical scenario. AI model thresholds were prespecified and set before the study using Lunit INSIGHT CXR.

RGT was defined as the interval, in minutes, between the time a radiologist opened a CXR for reporting and the time the report was finalized, as recorded in the PACS during session 1 or in the AI orchestration system during session 2. This metric reflects the active reporting time spent by the radiologist per study and serves as a direct measure of reporting efficiency.

TAT was defined as the interval, in minutes, between the time a radiologist started the reporting session and the time the report was finalized, as recorded in the PACS during session 1 or in the AI orchestration system during session 2. TAT therefore captures both waiting time and active reporting time within the reporting workflow. Thus, TAT is the sum of waiting time and RGT (Figure 4). This is a session-internal metric and differs from conventional TAT definitions in the radiology literature, which typically encompass the full interval from order placement or image acquisition to report delivery. As studies were reported within a fixed session of predetermined length, aggregate TAT per study also reflects session queue duration and should not be interpreted as equivalent to individual patient waiting time.

### Sample Size Calculations

On the basis of pilot data from 193 radiographs, a sample size was calculated to achieve 90% power with a 95% CI [22]. This calculation was performed to ensure sufficient precision in detecting differences in time-based workflow metrics between unaided and AI-assisted reporting sessions. The recommended sample size for the study was 960 radiographs.

### AI-Triage Classification

The AI-triage system was designed to triage films according to a categorical approach to CXR study classification, using a notification level scale of 1 to 4 (1=“normal,” 2=“nonurgent,” 3=“urgent,” and 4=“critical”), based on the presence of the 10 target findings, as validated by van Beek et al [23]. This categorization was used solely to support worklist prioritization and ordering, rather than to determine clinical diagnoses. Pneumoperitoneum and pneumothorax were deemed critical; cardiomegaly, consolidation, mediastinal widening, pleural effusion, and nodules were deemed urgent; and atelectasis, calcification, and fibrosis were deemed nonurgent. The reliability of this triage system has been prospectively validated at the same institution in a cohort of 20,944 CXRs, demonstrating area under the receiver operating characteristic curve (AUC) values of 0.91, 0.92, and 0.91 for normal, nonurgent, and urgent categories, respectively, with sensitivities ranging from 82% to 93% and specificities ranging from 91% to 99% across triage levels [24].

The resulting notification levels informed the sequence in which studies appeared on the reporting worklist. The current reference standard practice does not triage films prior to reporting by radiologists. Consequently, all studies in the unaided reporting session were processed in a FIFO manner without differentiation by urgency.

### Performance Evaluation

The evaluation in this study focused exclusively on workflow-related outcomes associated with the use of an AI-triaged worklist and AI-assisted report generation. Workflow impact was assessed using time-based metrics that reflect routine clinical reporting processes. RGT and TAT were selected as the primary outcome measures, as they directly capture radiologist reporting efficiency and overall reporting timeliness within the department.

All analyses were conducted on reports finalized by board-certified radiologists, with AI serving as a workflow support tool rather than as a diagnostic decision-maker. The role of the AI system in this study was limited to worklist prioritization and report generation assistance, consistent with its deployment in routine clinical practice.

### Data Collection Process

We collected 1054 CXRs for analysis. The films were acquired using digital radiography machines, including DigitalDiagnost C90 (Philips), DigitalDiagnost 3 (Philips), DigitalDiagnost 4 (Philips), and Arcoma Precision i5 (Arcoma AB) for emergency department (ED) films and Fujifilm FDR Go (Fujifilm Holdings Corp) for the outpatient and inpatient studies. These imaging systems reflect the institution’s routine clinical equipment used during the study period.

Exclusion criteria in both study arms included workflow interruptions and technical errors due to system downtime that resulted in inaccurate recording of TAT and failed data orchestration in the AI-assisted arm of the study. These exclusions were applied to ensure that recorded RGT and TAT values accurately reflected routine reporting workflows. Orchestration errors were related to nonstandard CXR projections not being processed by the AI system, such as lateral, oblique, and apical projections.

### Statistical Analysis

A 1-sided Wilcoxon signed-rank test was used to compare differences between session 1 (unaided) and session 2 (AI-assisted) for paired, time-based workflow metrics, including RGT and TAT. A 2-tailed Mann-Whitney *U* test was used to compare differences in median TAT between different emergency levels in session 1 (unaided). These analyses were performed to evaluate differences in reporting efficiency and timeliness across workflow conditions.

All statistical analyses were performed using Python (version 3.7.6; Python Software Foundation) with SciPy (version 1.4.1). Data visualizations were created using R software (version 4.3.3; R Foundation for Statistical Computing).

### Ethical Considerations

Ethics approval was obtained from the SingHealth centralized institutional review board (CIRB 2023/2280), and the requirement for patient consent was waived due to the observational nature of the study and the use of routinely collected clinical workflow data, which were assessed to pose minimal risk to patients. Informed consent was obtained from the radiologists participating in the study.

## Results

### Cohort Demographics

Patient demographics consisted of 1054 CXRs, with a sex distribution of 59.1% (n=623) male and 40.9% (n=431) female (Table 1) patients. The ethnic composition varied, including Chinese (n=662, 62.8%), Malay (n=288, 27.3%), Indian (n=60, 5.7%), and White or multiracial (n=44, 4.2%) individuals. Age ranged from <10 years to >90 years, with a mean age of 63.85 (SD 21.28) years. This demographic distribution was reflective of the institution’s routine clinical population undergoing chest radiography during the study period**,** based on internal institutional data from a sample of 20,944 patients.

**Table 1:** Demographic distribution and characteristics of chest radiograph data (N=1054).

Characteristics			Studies, n (%)
**Sex**			
	Male	623 (59.1)	
	Female	431 (40.9)	
**Ethnicity^a^**			
	White or multiracial	44 (4.2)	
	Chinese	662 (62.8)	
	Indian	60 (5.7)	
	Malay	288 (27.3)	
**Age group (y)**			
	0-10	2 (0.2)	
	11-20	50 (4.7)	
	21-30	69 (6.5)	
	31-40	71 (6.7)	
	41-50	63 (6)	
	51-60	113 (10.7)	
	61-70	196 (18.6)	
	71-80	234 (22.2)	
	81-90	199 (18.9)	
	>90	57 (5.4)	
**Triage class**			
	Normal	415 (39.4)	
	Nonurgent	169 (16)	
	Urgent	442 (41.9)	
	Critical	28 (2.7)	
**Department or ward**			
	Intensive care unit	5 (0.5)	
	Emergency	588 (55.8)	
	Inpatient	312 (29.6)	
	Outpatient	149 (14.1)	

^a^Investigator-observed ethnicity.

### RGT per Session

The median RGT reduced from 2 (IQR 1-4) minutes in the unaided session to 32 (IQR 13-67) seconds in the AI-assisted reading session, resulting in a reduction of 1.47 (95% CI 1.42-1.5; *P*<.001) minutes. This represented a 77.1% reduction in mean RGT associated with AI-assisted reporting workflows.

AI-assisted report generation resulted in a reduction in median RGT across all categories of notification level (Table 2), with the greatest absolute and proportional reduction observed in studies categorized as normal. Median RGT for normal CXRs decreased from 2 (IQR 1-3) minutes to 12 (IQR 8-21) seconds, corresponding to a 90% reduction (*P*<.001).

**Figure 5:**
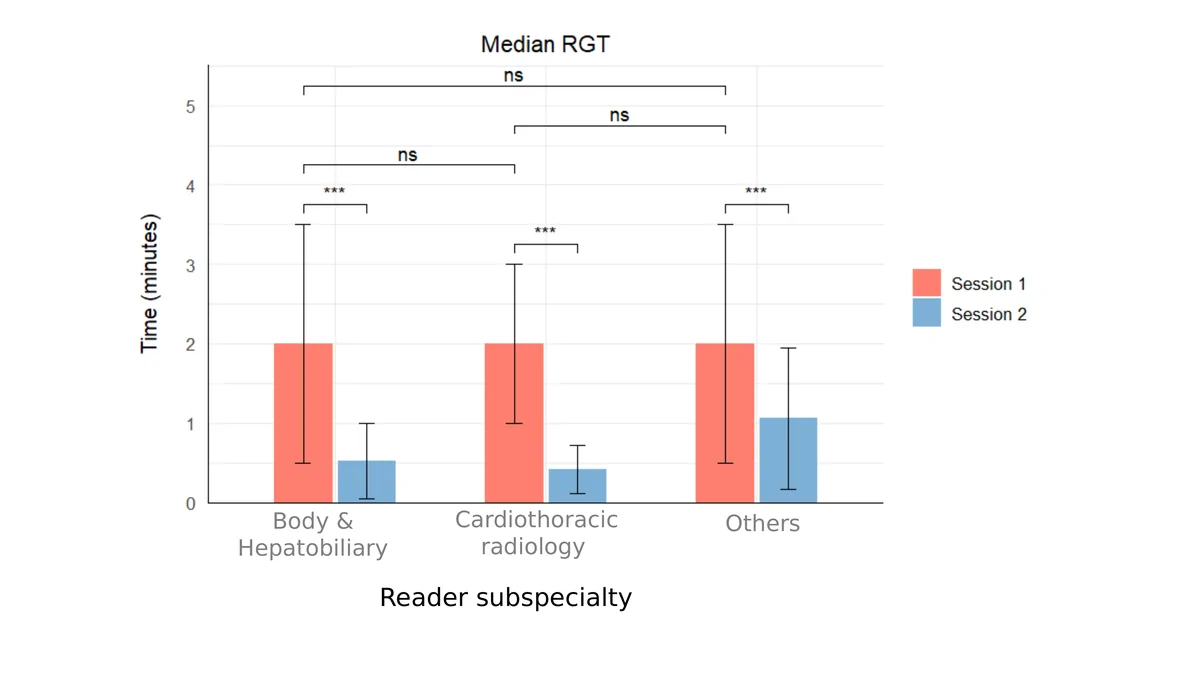
Comparison of median report generation time (RGT) across different groups of readers. ns: nonsignificant. ***P<.001.

A reduction in median RGT was also observed across all subspecialty groups of radiologists (Figure 5). For example, median RGT was reduced by 1.47 (95% CI 1.35-1.59) minutes, from 2 to 0.52 minutes (a reduction of 73.6%) for body and hepatobiliary radiologists; by 1.58 (95% CI 1.53-1.62) minutes, from 2 to 0.41 minutes (a reduction of 79.2%) for cardiac and chest radiologists; and by 0.94 (95% CI 0.68-1.48) minutes, from 2 to 1.05 minutes (a reduction of 47.1%) for other subspecialty radiologists (*P*<.001 for all 3 groups; Table S1 in Multimedia Appendix 1).

When stratified by the number of findings per study, reductions in median RGT were observed across all categories, with the greatest improvement observed in CXRs without any reported findings. In this group, median RGT decreased by 1.76 (95% CI 1.75-1.78) minutes, from 2 to 0.23 minutes, representing an 88.3% reduction (*P*<.001). For CXRs with 1 finding, median RGT decreased by 1.25 (95% CI 1.1-1.35) minutes, from 2 to 0.75 minutes (62.5% reduction; *P*<.001). For studies with 2 findings, median RGT decreased by 0.96 minutes (95% CI 0.83-1.97), from 2 to 1.03 minutes (48.3% reduction; *P*<.001). For studies with 3 or more findings, median RGT decreased by 0.85 (95% CI 0.51-2.02) minutes, from 2 to 1.14 minutes (42.9% reduction; *P*<.001; Table 2).

**Table 2:** Report generation time (RGT) for different notification levels.

Notification levels and sessions	Chest radiographs, n (%)	RGT (min), median (IQR)		Reduction in RGT (min), median (95% CI)	*P* value	Median reduction (%)
		Unaided	AI-assisted			
Overall	1054 (100)	2 (1-4)	0.53 (0.22-1.12)	1.47 (1.42 to 1.5)	<.001	73.3
Normal	415 (39.4)	2 (1-3)	0.2 (0.13-0.35)	1.8 (0.8 to 1.8)	<.001	90
Nonurgent	169 (16)	2 (1-3)	0.55 (0.33-0.98)	1.45 (1.33 to 1.52)	<.001	72.5
Urgent	442 (41.9)	2 (1-4)	0.92 (0.57-1.7)	1.08 (0.98 to 1.77)	<.001	54.2
Critical	28 (2.7)	3 (1.75-4.25)	1.93 (1.1-2.62)	1.07 (−0.31 to 2.33)	<.05	35.6

### TAT per Session

Mean TAT per study shortened from 876.21 (SD 1014.20; 95% CI 816.06-940.55) minutes to 82.25 (SD 83.40; 95% CI 76.98-87.27) minutes with AI assistance, representing a mean reduction of 793.97 (95% CI 733.02-860.91; *P*<.001) minutes. This translated to a 90.6% reduction in TAT for AI-assisted reporting compared to unaided sessions.

The effect of AI assistance on mean TAT reduction extended to all abnormal notification levels. Notably, for CXRs classified as critical (n=28), mean TAT decreased from 711.08 (95% CI 483.72-970.56) minutes to 41.56 (95% CI 27.1-57.79) minutes during AI-assisted sessions, representing a 94.1% reduction. This reduction was also observed at other noncritical notification levels: urgent, from 925.93 (95% CI 831.09-1028.21) minutes to 79.19 (95% CI 71.42-87.02) minutes, representing a 91.5% reduction; nonurgent, from 896.68 (95% CI 743.4-1051.2) minutes to 83.18 (95% CI 71.9-96.88) minutes, representing a 90.7% reduction; and normal, from 826.06 (95% CI 736.99-920.59) minutes to 87.87 (95% CI 79.61-96.47) minutes, representing an 89.4% reduction. The difference in TAT was statistically significant for all notification levels (*P*<.001 for all; Table 3). Notably, TAT improved across all triage categories, including normal and nonurgent studies, reflecting that AI-assisted report generation reduced per-study RGT across all categories and compressed the overall session duration rather than merely reordering a fixed workload.

**Figure 6:**
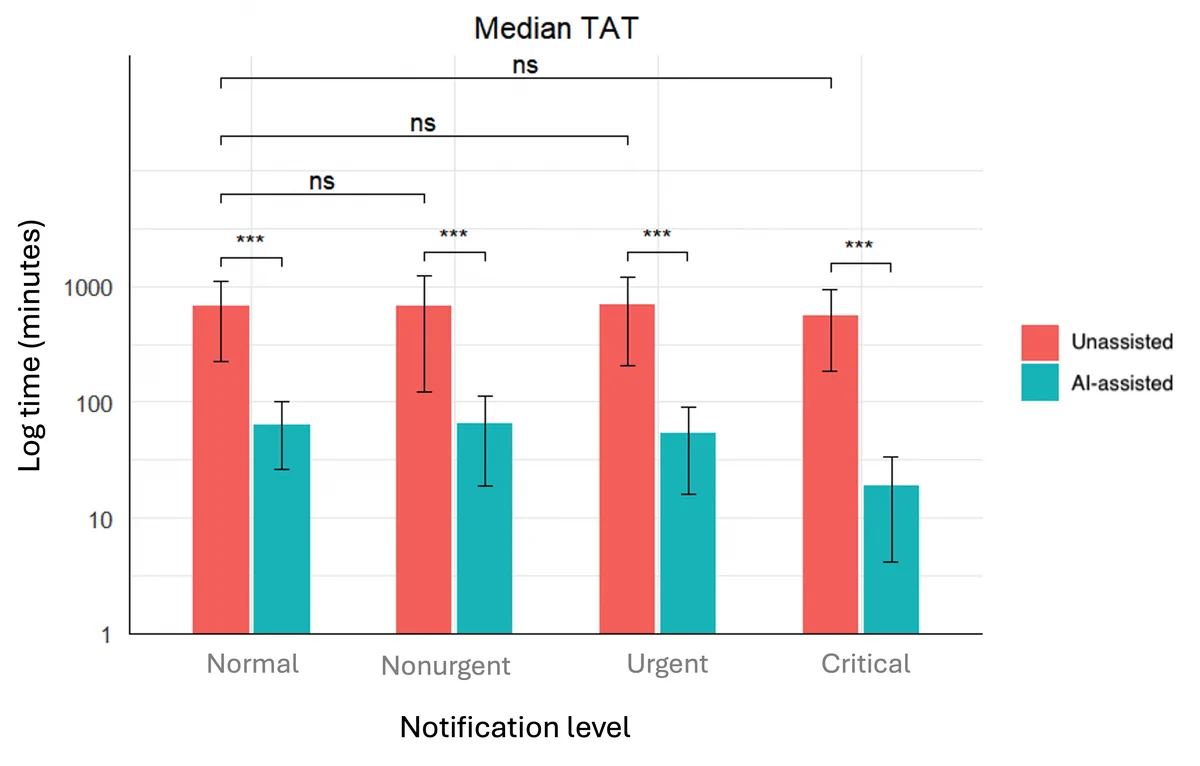
Reduction in median turnaround time (TAT) during the unassisted (session 1) and AI-assisted (session 2) reporting of chest radiographs. ns: nonsignificant. ***P<.001.

Figure 6 demonstrates the effect of AI triage on median TAT across notification levels. In the unaided reporting session, median TATs were not significantly different across notification categories (*P*>.05), reflecting the uniform processing of studies in a FIFO workflow. In contrast, during AI-assisted reporting, median TATs were differentiated by notification level, with critical studies reported substantially earlier than normal studies. Median TAT for critical studies decreased from 563.09 (IQR 210.6-968.04) minutes to 18.85 (IQR 14.89-44.31) minutes, compared with a decrease from 671.87 (IQR 98.39-989.91) minutes to 64.27 (IQR 34.81-110.24) minutes for normal studies (*P*<.001).

With AI assistance, median TAT was also reduced for each group of radiologists assessed according to subspecialty (Table S2 in Multimedia Appendix 1). Median TAT was reduced by 295.48 (95% CI 165.56-419.71) minutes, from 370.6 to 75.12 minutes (a reduction of 79.7%) for body and hepatobiliary radiologists; by 702.72 (95% CI 673.32-736.06) minutes, from 745.8 to 43.07 minutes (a reduction of 94.2%) for cardiac and chest radiologists; and by 516.02 (95% CI 469.09-616.05) minutes, from 595.13 to 79.1 minutes (a reduction of 86.7%) for other subspecialty radiologists. The difference in TAT was statistically significant for all subspecialty groups (*P*<.001 for all).

Waiting time, defined as the interval between the start of the reporting session and the opening of a study, decreased from a median of 687.1 (IQR 207.93-1157.42) minutes in the unaided session to 42.18 (IQR 16.78-93.87) minutes in the AI-assisted session, representing a 93.9% reduction.

**Table 3:** Turnaround time (TAT) for different notification levels.

Notification levels and sessions	Chest radiographs, n (%)	TAT (min), median (IQR)		Reduction in TAT (min), median (95% CI)	*P* value	Median reduction (%)
		Unaided	AI-assisted			
Overall	1054 (100)	675.87 (176.19-1164.17)	55.87 (29.87-107.22)	620 (567.38-656.62)	<.001	91.7
Normal	415 (39.4)	671.87 (98.39-989.91)	64.27 (34.81-110.24)	607.6 (508.44-649.72)	<.001	90.4
Nonurgent	169 (16)	683.58 (130.65-1251.07)	65.3 (28.77-121.42)	618.28 (500.12-686.97)	<.001	90.5
Urgent	442 (41.9)	699.9 (254.02-1239.95)	53.22 (24.8-99.15)	646.68 (570.24-688.59)	<.001	92.4
Critical	28 (2.7)	563.09 (210.6-968.04)	18.85 (14.89-44.31)	544.24 (232.28-853.41)	<.001	96.7

## Discussion

### Principal Findings

This study aimed to evaluate the impact of an AI-triaged worklist and AI-assisted report generation on radiologist workflow efficiency in a real-world hospital setting. Using a prospective paired crossover design, we demonstrated substantial reductions in both RGT and TAT for CXRs. Median RGT decreased by 73.3%, from 2 to 0.53 minutes, and mean TAT decreased by 90.6%, from 876.21 to 82.25 minutes. Critically, the AI-assisted workflow introduced urgency-differentiated reporting that was entirely absent in the unaided FIFO condition; median TAT for critical studies fell from 563.09 to 18.85 minutes, while TAT did not differ significantly across urgency categories during the unaided session. These findings directly address the study’s stated objectives and provide real-world evidence of the operational value of AI-integrated radiology workflows.

### Impact of AI on RGT and Radiologist Efficiency

AI-assisted report generation improved documentation efficiency and workflow support without altering diagnostic decision-making. This finding parallels results from other studies that have reported reductions in reporting time with AI-assisted workflows. Yacoub et al [25] demonstrated a 22.1% reduction in chest computed tomography RGT. In the interpretation of lumbar spine stenosis on magnetic resonance imaging (MRI), Lim et al [26] demonstrated a reduction in mean interpretation time per study from 124 to 274 seconds to 47 to 71 seconds, with equivalent or improved interobserver agreement. A systematic review by Li et al [27] also showed improved detection, diagnostic performance, and AUC in readers assisted by AI-based devices.

The largest reduction in RGT was achieved in normal notification level studies, a finding similar to that of other studies that found the greatest impact of AI on reducing normal RGT [28]. This could be attributed to several factors, including the increased efficiency of finalizing a normal report template produced by the report generation tool. Additionally, AI concordance with normal studies could speed up the decision-making process by supporting radiologists’ true-negative judgment for a normal study, lowering the threshold for finalizing a normal report [24].

Although this study was not designed to formally assess diagnostic accuracy or report quality, the concordance analysis provides indirect evidence of maintained clinical vigilance during AI-assisted reporting. In 46.7% (n=492) of cases, radiologists removed at least 1 AI-suggested finding, demonstrating active critical appraisal rather than passive adoption of AI output. In 3.1% (n=33) of cases, radiologists added findings not flagged by the AI, confirming continued independent diagnostic assessment. All reports were finalized by board-certified radiologists, and the AI report generation tool required explicit acceptance or rejection of each suggested finding before finalization. Formal evaluation of downstream clinical outcomes, including report completeness, clinical action rates, and patient outcomes, was beyond the scope of this study, and we recommend this as a priority for future research.

### Impact of AI-Assisted Workflow and Triage in Clinical Practice

The feasibility of AI-assisted triaging systems in radiology practice has been explored, for example, in workflows where time-critical radiological findings are key components of the treatment algorithm, such as acute ischemic stroke [29]. In this study, the impact of AI assistance on TAT was more pronounced than the reduction observed in RGT.

This improvement in TAT is likely due to multiple overlapping efficiencies introduced in the AI-assisted workflow. These include the automated prioritization of high-urgency studies (triage); the use of structured report templates, which reduced documentation time; and the minimization of administrative delays through direct worklist assignments. Although the relative contribution of each component could not be isolated, their combined effect resulted in a measurable reduction in overall reporting TAT. Future studies may consider factorial designs to quantify the impact of each component independently.

As studies were reported on a FIFO basis, studies of varying urgency experienced similar reporting delays in the unaided workflow. In contrast, the AI-assisted workflow ensured that urgent and critical studies were reported earlier according to priority, with the remaining studies reported thereafter.

The European Society of Thoracic Imaging highlights the importance of integrating AI algorithms into existing departmental workflows [30]. The productivity gains exhibited in this study should be interpreted within the specific institutional context, and it is unclear whether our findings regarding the reduction in TAT for normal and nonurgent studies will be generalizable across different institutions with varying workflows and infrastructure. Hwang et al [31], for example, demonstrated no significant difference in workflow efficiency using AI-assisted interpretation of CXRs. Departments with a smaller radiology workforce may choose to deprioritize the reporting of AI-triaged normal and nonurgent studies, depending on the radiologist resources available. This would result in longer TATs for normal and nonurgent studies.

Furthermore, improved TAT in the context of AI-augmented CXR worklists is not generalizable across all modalities. For example, Wenderott et al [32] showed an increase in throughput time using AI-assisted detection for prostate MRI compared to a non–AI-augmented workflow, highlighting the importance of modality-specific evaluation of AI-assisted workflow interventions.

In our setting, the AI-assisted triage system prioritized cases across a diverse patient mix, including ED (n=588, 55.8%), inpatient (n=312, 29.6%), and outpatient (n=149, 14.1%) frontal CXRs. Critical notifications were predominantly flagged among ED and inpatient cases, while most normal studies originated from outpatient or postdischarge imaging. The AI-driven ordering ensured that critical inpatient studies were surfaced immediately, even when embedded in high-volume mixed worklists. By prioritizing urgent studies within mixed worklists, the AI-assisted workflow enabled earlier reporting of time-sensitive examinations without altering final reporting responsibility. This operational shift reflects a real-world enhancement in radiologist workflow efficiency, especially in high-throughput environments where FIFO models are inadequate.

### Limitations and Future Research

This study was conducted at a single, large, fully staffed academic institution, which limits the generalizability of the findings. Departments with smaller radiology workforces or different PACS configurations may experience attenuated benefits from AI-based worklist prioritization, as the ability to act on triage signals immediately is contingent on available staffing. Furthermore, as AI-triaged worklists and AI-assisted report generation were introduced simultaneously, the relative contributions of each component to the observed efficiency gains could not be isolated. Future factorial study designs could enable independent quantification of their effects. Multicenter studies across varied clinical environments are needed to validate these findings more broadly.

The critical triage subgroup comprised only 28 studies, resulting in wide CIs for TAT reduction estimates (95% CI 232-853) and limited statistical power. These findings should therefore be interpreted with caution and validated in larger cohorts. Additionally, the within-reader, within-case design required radiologists to report the same studies on 2 separate occasions, which is inherently artificial and does not replicate the natural single encounter characteristic of routine clinical practice. Furthermore, all radiologists were required to use the AI report generation tool in the second session to ensure standardized data collection. This does not reflect real-world practice, where tool adoption is voluntary, and the observed RGT reduction may therefore partly reflect this workflow constraint rather than the intrinsic benefit of AI assistance.

The sample size calculation was based on aggregate pilot data and did not explicitly account for reader clustering effects or the skewed distribution of temporal outcomes. Future studies should incorporate these factors into power calculations for paired workflow designs. Additionally, waiting time data were available only at the aggregate session level and could not be stratified by triage category, limiting the precision with which the impact of AI prioritization on time-sensitive studies could be demonstrated. Furthermore, our TAT metric captures the session-internal interval from worklist initiation to report finalization and does not encompass preimaging steps. Direct numerical comparisons with published TAT benchmarks using conventional order-to-report definitions should therefore be interpreted with caution.

Finally, this study evaluated only immediate, short-term workflow efficiency outcomes. Patient-level end points, such as length of stay, readmission rates, and downstream clinical decisions, were not assessed, nor was a cost-benefit analysis performed. Future studies should examine whether improvements in reporting timeliness translate into measurable clinical benefits and evaluate the economic sustainability of AI workflow integration at scale. The impact of AI-assisted reporting on diagnostic accuracy and report discrepancy rates was also not assessed, and future work should evaluate whether such workflows maintain or improve radiologist diagnostic performance compared to unaided reading.

### Conclusions

The findings of this study demonstrate that integrating AI-based worklist triage and report generation support into radiology workflows can produce meaningful improvements in reporting efficiency without altering the clinical responsibility of the radiologist. Beyond the time savings observed, the more fundamental shift is the transition from urgency-blind, FIFO-based reporting to urgency-aware, prioritized workflows, a change with direct implications for patient safety and the timeliness of clinical decision-making.

As AI tools become increasingly embedded in health care infrastructure, this study contributes real-world evidence that their value extends beyond diagnostic augmentation to systematic workflow redesign. For radiology departments operating at scale, AI-driven prioritization represents a pragmatic, implementable intervention to address reporting backlogs and reduce delays for time-sensitive findings. Future work should examine whether these efficiency gains translate into measurable downstream clinical outcomes and evaluate their scalability across institutions with varying staffing models and imaging volumes.

## Appendix

Multimedia Appendix 1 Comparisons of report generation time and turnaround time between subspecialty groups of radiologists.

## Abbreviations

AUC: area under the receiver operating characteristic curve
CIRB: centralized institutional review board
CXR: chest radiograph
ED: emergency department
FIFO: first-in, first-out
MRI: magnetic resonance imaging
PACS: picture archiving and communication system
RGT: report generation time
SPIRIT: Standard Protocol Items: Recommendations for Interventional Trials
TAT: turnaround time

## Data Availability

The datasets generated and analyzed during the current study are not publicly available due to institutional and patient privacy restrictions but may be made available by the corresponding author on reasonable request and subject to institutional approval.
