# Supplementary material for: Impact of AI-Triaged Worklists and AI-Assisted Report Generation on Radiology Turnaround Times: Prospective Real-World Study
Source: J Med Internet Res. 2026 Aug 31;28:e92181. doi: 10.2196/92181 (PMC13575548; doi:10.2196/92181)
Supplement: Supplementary file 1 [file jmir_v28i1e92181_app1.docx]

**Supplementary Table 1:** Comparison of report generation time between sub-specialty groups of radiologists

| **Reader subspecialty** | **Session** | **N** | **Mean**  **[95% CI]** | **SD** | **Reduction in mean RGT [95% CI]** | **Median (IQR)** | **Reduction in median RGT [95% CI]** | ***P* value** | **Mean RGT reduction (%)** | **Median RGT reduction (%)** |
| --- | --- | --- | --- | --- | --- | --- | --- | --- | --- | --- |
| Body and hepatobiliary | Unaided | 176 | 6.00  [4.09-8.63] | 16.35 | 5.09  [3.15-7.70] | 2.00  (1.00-4.00) | 1.47  [1.35-1.59] | < 0.001 | 84.8 | 73.8 |
|  | Aided |  | 0.91  [0.75-1.09] | 1.14 |  | 0.52  (0.23-1.18) |  |  |  |  |
| Cardiac and chest radiology | Unaided | 610 | 3.98  [3.44-4.56] | 7.19 | 3.38  [2.85-3.97] | 2.00  (1.00-3.00) | 1.58  [1.53-1.62] | < 0.001 | 85.1 | 79.2 |
|  | Aided |  | 0.59  [0.55-0.65] | 0.60 |  | 0.41  (0.18-0.78) |  |  |  |  |
| Others | Unaided | 268 | 5.07  [3.79-6.85] | 12.42 | 2.88  [1.53-4.67] | 2.00  (1.00-4.00) | 0.94  [0.68-1.48] | < 0.001 | 56.9 | 47.1 |
|  | Aided |  | 2.18  [1.77-2.63] | 3.52 |  | 1.05  (0.45-2.23) |  |  |  |  |

**Supplementary Table 2:** Comparison of turnaround time between sub-specialty groups of radiologists

| **Reader subspecialty** | **Session** | **N** | **Mean [95% CI]** | **SD** | **Reduction in mean TAT [95% CI]** | **Median (IQR)** | **Reduction in median TAT [95% CI]** | ***P* value** | **Mean TAT reduction(%)** | **Median TAT reduction (%)** |
| --- | --- | --- | --- | --- | --- | --- | --- | --- | --- | --- |
| Body and hepatobiliary | Unaided | 176 | 467.99 [406.67-524.91] | 396.88 | 392.25 [329.94-450.28] | 370.60 (106.70-743.36) | 295.48 [165.56-419.71] | < 0.001 | 83.8 | 79.7 |
|  | Aided |  | 75.73 [69.94-81.52] | 39.14 |  | 75.12 (45.62-109.21) |  |  |  |  |
| Cardiac and chest radiology | Unaided | 610 | 940.82 [857.71-1025.94] | 1032.04 | 863.04 [775.94-949.52] | 745.80 (137.52-1340.93) | 702.72 [673.32-736.06] | < 0.001 | 91.7 | 94.2 |
|  | Aided |  | 77.77 [70.52-85.06] | 94.64 |  | 43.07 (26.40-98.62) |  |  |  |  |
| Others | Unaided | 268 | 997.24 [860.72-1146.63] | 1178.94 | 900.54 [760.08-1052.98] | 595.13 (352.86-987.41) | 516.02 [469.09-616.05] | < 0.001 | 90.3 | 86.7 |
|  | Aided |  | 96.69 [87.49-106.67] | 75.51 |  | 79.10 (41.56-125.00) |  |  |  |  |
